# Inhibitory effect of 2-methyl-naphtho[1,2,3-de]quinolin-8-one on melanosome transport and skin pigmentation

**DOI:** 10.1038/srep29189

**Published:** 2016-07-06

**Authors:** Jong il Park, Ha Yeon Lee, Ji Eun Lee, Cheol hwan Myung, Jae Sung Hwang

**Affiliations:** 1 Department of Genetic engineering & Graduate School of Biotechnology, College of Life Sciences, Kyung Hee University, Gyeonggi-do 446-701, Republic of Korea

## Abstract

Melanosomes are lysosome-related organelles with specialized capabilities of melanin synthesis and movement mediated by the Rab27a-Melanophilin-MyosinVa protein complex. In this study, we found that 2-methyl-naphtho[1,2,3-de]quinolin-8-one (MNQO) induced melanosome aggregation around the nucleus in melan-a melanocytes and in melan-a melanocytes/SP-1 keratinocyte co-cultures without inducing toxicity or changing the melanin content. Western blot and real-time PCR analyses showed that MNQO decreased expression of the Rab27a, Melanophilin and MyosinVa proteins and mRNAs, respectively, in melan-a melanocytes. In a reconstituted human epidermis model, treatment with 0.001% MNQO reduced skin pigmentation. Also, MNQO reduced skin pigmentation in brown guinea pigs induced by UVB irradiation. These results indicated that regulation of melanosome transport may serve as a good target for new skin depigmenting agents and MNQO itself could be a candidate.

In mammalian hair and skin, melanogenesis proceeds within melanosomes, which are specialized lysosome-related vesicles in melanocytes of the basal layer of the epidermis^1^. The three principal regulatory enzymes in melanin synthesis are tyrosinase, tyrosinase-related protein-1(TRP-1) and TRP-2^2^. After synthesis and maturation in the central region of the cell, melanosomes are transported by tubulin- and actin-dependent motor proteins along the cytoskeleton to the cell periphery and tips of the melanocyte dendrites^3^. Following transfer to surrounding keratinocytes, melanosomes form the melanin caps that protect against UV-induced DNA damage and establish the pattern and intensity of pigmentation in human epidermal melanocytes^4^. Melanosomes undergo both a bidirectional microtubule-dependent transport and a unidirectional actin-dependent transport that allows melanosomes to anchor at the dendrite tips^5^.

The mechanism of melanosome transport from the perinuclear area to dendrite tips involves signaling by the small GTPase Rab27a, and its effector molecule Slac2-a/Melanophilin (Mlph), which, following recruitment of the actin-dependent motor protein MyosinVa (MyoVa), allows melanosomes to move on the actin network^6^,^7^,^8^,^9^,^10^. Mutations in genes encoding the Rab27a, Mlph and MyoVa proteins result in formation of an abnormal tripartite complex that impairs melanosome transport and allows melanosomes to aggregate in the perinuclear region^11^,^12^. In humans, a melanosome transport defect leads to Griscelli syndrome (GS), an autosomal recessive disorder characterized by hypopigmentation in the skin and hair^13^. Mutation in MyoVa is associated with GS type I, characterized by neurological impairment, while mutation in Rab27a is linked to GS type II, with immunological impairment. In GS type III, linked to mutation in Mlph, hypopigmentation occurs without other clinical features^14^,^15^. Melanosome transport defects in the mouse include *dilute* (MyoVa), *ashen* (Rab27a) and *leaden* (Mlph)^16^,^17^.

2-methyl-naphtho[1,2,3-de]quinolin-8-one (MNQO) is a natural compound extracted from the bark of the South American lapacho tree (Tabebuia avellanedae [Bignoneaceae]) (Fig. 1). A traditional medicine known as *Ipê-Roxo* is prepared from this tree in South America, and in both South and North America similar preparations have been applied as antineoplasic, antifungal, antiviral, antimicrobial, antiparasitical and anti-inflammatory treatments^18^. In the present study, we found that MNQO inhibited melanosome transport in melanocytes, skin pigmentation in reconstituted human epidermis and brown guinea pig *in vivo*. We also investigated the inhibitory mechanism of MNQO on melanosome transport in melanocytes.

## Results

### Effect of MNQO on the perinuclear localization of melanosomes and melanin synthesis in melan-a melanocytes

**Figure 2:**
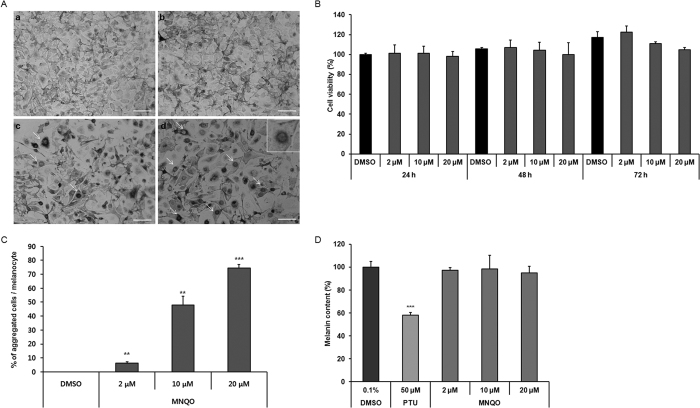
(**A**) Melanosome aggregation in melan-a melanocytes treated with MNQO. Bright-field image (x200) of melanocytes treated with 0.1% DMSO (a), 2 μM MNQO (b), 10 μM MNQO (c) and 20 μM MNQO (d) for 72 h. Melanosome aggregation in the MNQO-treated cells is indicated by white arrows (scale bar = 20 μm). (**B**) Effects of MNQO on growth of melan-a melanocytes. Cell viability was measured using a cytotoxicity assay (see Methods). Melan-a melanocytes were treated with 2 μM, 10 μM and 20 μM MNQO, or with 0.1% DMSO (control) for 24 h. Optical density was measured at 450 nm. Values are presented as the mean ± SD from three determinations (n = 3). (**C**) Percentage of melanosome aggregated cells in melan-a melanocyte cultures. After treatment for 72 h with each compound, the number of aggregated cell was counted in each culture. Evaluation of melanosome aggregation was performed by counting cells with perinuclear melanosome aggregates in three random microscopic fields per well at x200 magnification. Values are presented as the mean ± SD from three wells (n = 3). Data were analyzed by Student’s unpaired *t-*test. **p < 0.01; ***p < 0.001. (**D**) Melanin content of melan-a melanocytes incubated with MNQO at 2 μM, 10 μM and 20 μM for 3 days. Fifty μM PTU was used as a positive control treatment. Values are presented as mean ± SD from three determinations (n = 3). Data were analyzed using Student’s unpaired *t-*test, ***p < 0.001.

In untreated melan-a melanocytes, the melanosomes were distributed throughout the cell body (Fig. 2A). Following treatment with MNQO at 2 μM, 10 μM and 20 μM for 72 h, melanosomes were shown to cluster in the perinuclear region of the cells. The cells and dendrites did not change in shape, however, and MNQO at these concentrations for 72 h did not affect of cell viability of melan-a melanocytes (Fig. 2B).

After treatment for 72 h with MNQO, the number of cells with perinuclear melanosome aggregation was counted as described in the Methods section. The melanosome aggregation values were 6.5 ± 0.7% at 2 μM, 48.1 ± 6.1% at 10 μM and 74.6 ± 2.4% at 20 μM MNQO (Fig. 2C). Treatment with MNQO at 10 μM and 20 μM significantly increased the proportion of cells per culture well showing perinuclear melanosome aggregation.

To test the effects of MNQO treatment on melanin synthesis in melan-a melanocytes, melanin content was measured in cells treated with MNQO at 2 μM, 10 μM and 20 μM for 72 h (Fig. 2D). Phenylthiourea was used as a positive control^19^. MNQO treatment did not reduce melanin content in melan-a melanocytes. Therefore, MNQO induced perinuclear melanosome aggregation, but did not change melanin content in melan-a melanocytes.

### Effect of MNQO on melanosome localization in a cell co-culture system

**Figure 3:**
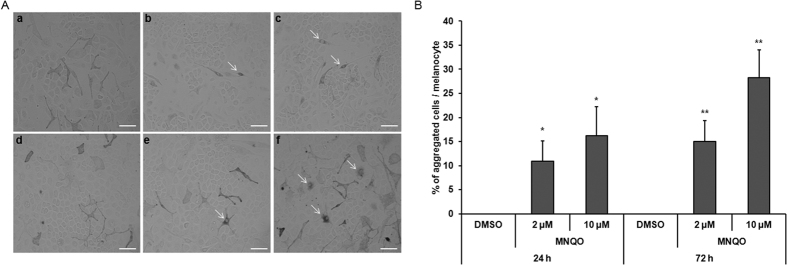
Effects of MNQO on melanosome localization in co-culture. Melan-a melanocytes and SP-1 keratinocytes were co-cultured with 2 μM or 10 μM MNQO, or with 0.1% DMSO, for 24 h or 72 h. (**A**) Images are shown for co-cultures treated with 0.1% DMSO (a), 2 μM MNQO (b) and 10 μM MNQO (c) for 24 h; and with DMSO (d), 2 μM MNQO (e) and 10 μM MNQO (f) for 72 h before the cultures were photographed. The MNQO inhibited melanosome transport in melanocytes and keratinocytes in co-culture (white arrows) (scale bar = 20 μm). (**B**) Percentage of melanosome aggregated cells per total melanocytes in co-culture. After treatment for 24 h and 72 h, the number of aggregated cells was counted in each culture. Evaluation of melanosome aggregation was performed by counting cells with perinuclear melanosome aggregates in three random microscopic fields per well at x200 magnification. Values are presented as the mean ± SD from three wells (n = 3). Data were analyzed by Student’s unpaired *t-*test. *p < 0.05; **p < 0.01.

To assemble this model of human skin, melan-a melanocytes and SP-1 keratinocytes were seeded and cultured together. Treatments with MNQO were performed at 2 μM and 10 μM for 24 h and 72 h (Fig. 3A). Compared to untreated cells in co-culture, co-cultured melanocytes treated with MNQO showed significant time-dependent melanosome aggregation in the perinuclear region and significantly increased the proportion of melanosome aggregation (Fig. 3B and Table 1). Therefore, MNQO inhibited melanosome transport in this melanocyte-keratinocyte co-culture system.

### Effect of MNQO on the expression of Rab27a, Mlph and MyoVa proteins

**Figure 4:**
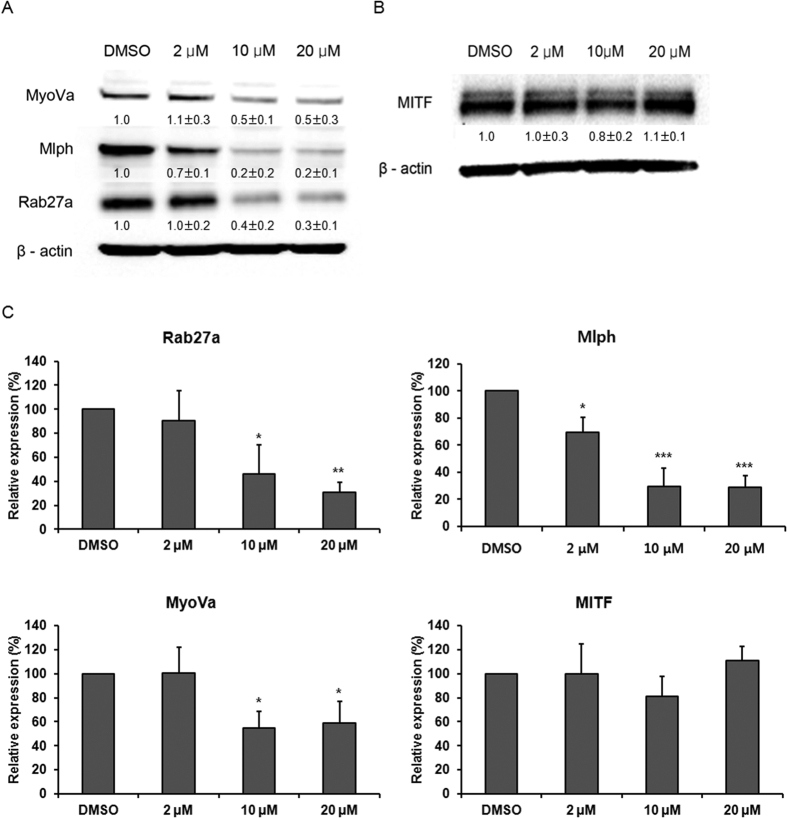
Inhibition by MNQO of tripartite complex proteins that mediate melanosome transport. Levels of Rab27a, Mlph and MyoVa (**A**) and MITF proteins (**B**) were measured in melanocytes by western blotting. The melan-a melanocytes were treated with 2 μM, 10 μM and 20 μM MNQO, or with 0.1% DMSO for 72 h. (**C**) Quantification of Rab27a, Mlph, MyoVa and MITF protein expression level. Values are mean value of expression normalized to β-actin from three independent experiments ± SD.

To identify the effect of MNQO on expression of tripartite complex proteins, we performed western blotting. Melan-a melanocytes were treated with MNQO at 2 μM, 10 μM and 20 μM for 72 h. Following treatment with MNQO for 72 h, levels of Rab27a, Mlph and MyoVa proteins in melan-a melanocytes were dose-dependently reduced as compared to untreated control cells (Fig. 4A,C). In the present report, MITF regulates transcription of Rab27a as a transcription factor in melanoma cells^5^. We identified the expression of MITF protein, a known upstream regulator of Rab27a, but the level of MITF protein was not changed (Fig. 4B,C).

### Effect of MNQO on expression of Rab27a, Mlph and MyoVa mRNAs

**Figure 5:**
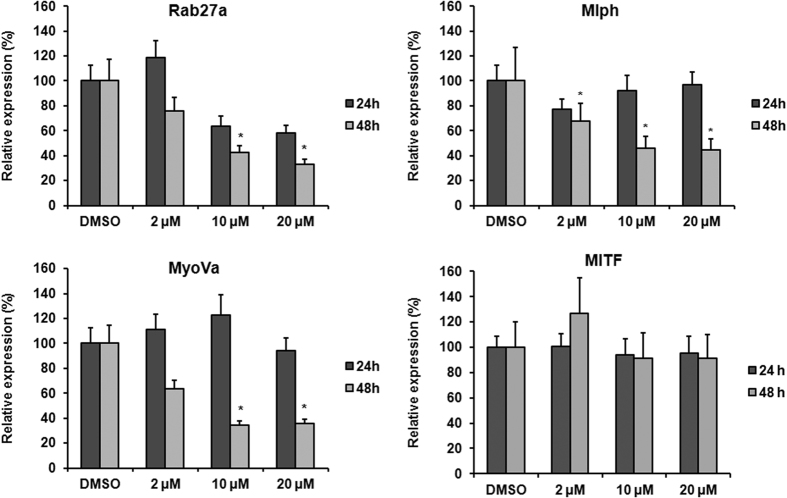
Effects of MNQO on Rab27a, Mlph, MyoVa and MITF mRNA expression level in melanocytes. Levels of Rab27a, Mlph, MyoVa and MITF mRNA were measured in melan-a melanocytes by real-time PCR analysis. The melan-a melanocytes were treated with MNQO at 2 μM, 10 μM and 20 μM, or with 0.1% DMSO for 24 h and 48 h. Values are mean value of expression normalized to β-actin and relative to control from three independent experiments ± SD. Data were analyzed using the Student’s unpaired *t-*test. *p < 0.05; **p < 0.01.

To investigate the effect of MNQO on expression of tripartite complex proteins at the level of transcription, we performed real-time PCR using specific primers for Rab27a, Mlph and MyoVa. Melan-a melanocytes were treated with MNQO at 2 μM, 10 μM and 20 μM for 24 h and 48 h (Fig. 5). Following 24 h treatment, no changes in expression of Rab27a, Mlph or MyoVa mRNAs were observed. After 48 h treatment, dose-dependent reductions in Rab27a, Mlph and MyoVa mRNAs became apparent, but MITF mRNAs was not changed. Thus MNQO may inhibit melanosome transport by inhibiting Rab27a, Mlph and MyoVa gene transcription by unknown upstream regulator, not MITF.

### Effect of MNQO on melanin production in a reconstituted human skin model

**Figure 6:**
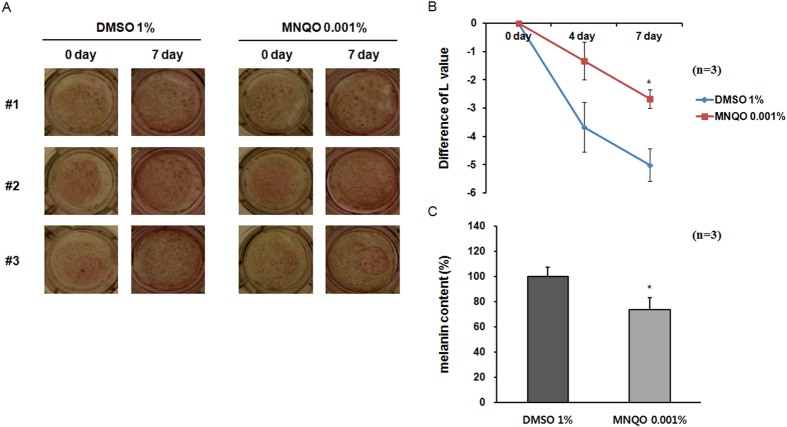
Inhibition of melanin production in a reconstituted human skin model by treatment with MNQO. (**A**) Photographs of melanoderm cultures and (**B**) L-value data. (**C**) The melanin content of the lysates was measured as optical density at 490 nm after solubilizing proteins from reconstituted human skins with 1 N NaOH (n = 3). Pigmented skin equivalents were treated for 7 days with the indicated compounds. Data were analyzed using Student’s unpaired *t* test. *p < 0.05.

Reconstituted human skin consisted of normal human-derived epidermal keratinocytes (NHEK) and melanocytes (NHEM). When we culture reconstituted human skin *in vitro*, NHEM undergoes melanogenesis and leads pigmentation with time. During treatment with 0.001% MNQO, pigmentation progressed more slowly and showed lower intensity after 7 days than in 1% DMSO-treated control skin (Fig. 6A). Reduced induction of pigmentation by MNQO treatment compared with DMSO control was quantified by L-values (Fig. 6B). Melanin assays revealed a significant reduction of melanin content in MNQO treated reconstituted human skins compared with the 1% DMSO control (Fig. 6C).

### Effect of MNQO on UVB-induced skin pigmentation in brown guinea pigs

**Figure 7:**
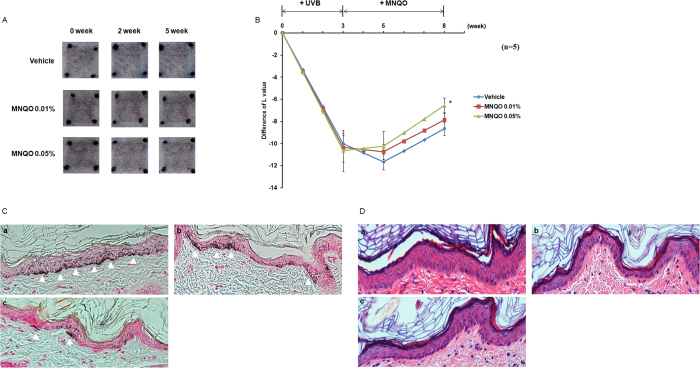
Effect of MNQO on skin pigmentation in UVB-induced brown guinea pigs. (**A**) Photographs of the lightening effects of MNQO on UVB-induced brown guinea pigs and (**B**) L-value data. Data were analyzed using Student’s unpaired *t* test (*p < 0.05). (**C**) Biopsy specimens from the vehicle (a), 0.01% and 0.05% MNQO-treated area (b,c) after 5 weeks of topical application examined for Fontana-Masson staining and (**D**) H&E staining.

To confirm the effect of MNQO on UVB-induced skin pigmentation, we used *in vivo* brown guinea pig models as described in method section 2.10. A reduction of pigmentation was observed in the MNQO-treated skin area compared to vehicle-treated area (Fig. 7A). A significant reduction of pigmentation was observed by 0.05% MNQO treatment at 5 weeks after UVB irradiation (Fig. 7B). Tissue biopsies were analyzed by Fontana-Masson and H&E staining (Fig. 7C,D). Accumulation of melanin was induced by UVB irradiation in the basal layer of epidermal in vehicle-treated areas (arrow head), compared with reduced melanin synthesis in 0.05% MNQO-treated areas. Any histological changes were not observed by H&E stained skin section.

## Discussion

In transport of melanosomes from the perinuclear region of a cell to the dendrite tips, a “melanosome transport complex,” consisting primarily of Rab27a, Mlph and MyoVa proteins, is involved. Rab27a, serving as a transporter protein, binds to the melanosome surface and engages in actin-dependent melanosome movement through direct interaction with the effector molecule Mlph and indirect interaction with exon F-containing isoforms of MyoVa^20^. All three proteins are essential for normal melanosome transport to the periphery of the cell. A defect in any one of them perturbs melanosome transport and ultimately affects skin pigmentation, without any change in melanin synthesis. As a result, skin coloring may be patchy or pale.

To identify compounds that inhibit melanosome transport, we tested a variety of plant derivatives, and MNQO appeared to induce melanosome aggregation within melan-a melanocytes. We observed that MNQO inhibited melanosome transport in monolayer cultures of melan-a melanocytes and in co-cultures consisting of SP-1 keratinocytes and melan-a melanocytes. Significant clustering of melanosomes in perinuclear areas was shown in cells treated with 10 μM and 20 μM MNQO as compared to control melan-a melanocytes. It was reported that close associations exist between melanocytes and their neighboring keratinocytes, and these two cell types together compose the epidermal melanin unit^21^. Keratinocytes receive melanin from the melanocytes via endo/exocytosis, and skin coloring develops through this interaction^22^. Melanosome clustering was also observed in a co-culture system composed of SP-1 keratinocytes and melan-a melanocytes treated with 10 μM MNQO. Melanosomes transfers to SP-1 cells could be reduced by MNQO in co-culture systems by clustered melanosome in melan-a cells. To test the effect of MNQO on melanin production, we treated melan-a melanocytes with MNQO at 2 μM, 10 μM and 20 μM for 72 h; however, none of these treatments affected melanin synthesis detectably. Using Western blot analysis, we found that Rab27a, Mlph and MyoVa protein levels declined significantly with MNQO treatment. Analysis by RT-PCR demonstrated a graded reduction of Rab27a, Mlph and MyoVa mRNA expression in co-cultures treated for 48 h with 2 μM, 10 μM and 20 μM MNQO. Real-time PCR also revealed reductions in all three mRNAs after 48 h treatment with MNQO at 2 μM, 10 μM and 20 μM. These results suggested that MNQO decreased melanosome transport in melanocytes through the inhibition of Rab27a, Mlph and MyoVa expression at the level of gene transcription. When we checked the expression of MITF protein and mRNA, a known transcriptional factor of Rab27a, the level of MITF protein and mRNA were not changed. Therefore, MNQO could inhibit Rab27a, Mlph and MyoVa gene expression by affecting unknown upstream regulator. It was reported that augmented STAT3 activation decreased gene expression of Rab27a in neutrophils^23^. However, there was no report on the role of STAT3 on Rab27a expression in melanocyte. Further studies on the effect of MNQO on STAT3 activation are needed. The upstream regulator of Mlph or MyoVa gene expression has not been identified. Also, common regulatory factor of three genes has not been reported yet. Therefore, it will be helpful for understanding the regulatory mechanism of melanosome transport and vesicle transport if we can identify the upstream regulator of these genes.

Manassantin B and Hesperidin have been reported as an inhibitor of melanosome transport^24^,^25^. Manassantin B disrupts Mlph-MyoVa interaction and inhibits melanosome transport. Hesperidin blocks the Rab27a-Mlph interaction *in silico* without inhibiting melanin synthesis or directly inhibiting tyrosinase. Although these two compounds could inhibit melanosome transport by reducing the interaction of protein-protein binding, but MNQO inhibited melanosome transport by decreasing the Rab27a, Mlph and MyoVa gene expression and it does not affect melanin synthesis in melanocytes.

Finally, we investigated the effect of MNQO in reconstituted human skin maintained *in vitro* and in brown guinea pigs *in vivo*. Treatment of MNQO reduced skin pigmentation in reconstituted human skin and brown guinea pigs, without any side effects such as toxicities.

Many compounds have been developed for skin whitening or treating pigmentation disorders^26^. Most of these substances decreased melanin production by inhibition of tyrosinase, but they have not been used in clinic for efficacy or safety issues. Recently, Rhododendrol, a tyrosinase inhibitor was developed for skin whitening cosmetics^27^. However, rhododendrol was identified to trigger a depigmentary disorder, leukoderma and products containing rhododendrol were withdrawn in the market. Therefore, it is necessary to develop differentiated compounds for skin whitening or treating pigmentation disorders.

Controlling melanosome transport, such as Mlph, could be more efficient and safe way to modulate skin pigmentation. The capacity of MNQO to reduce skin pigmentation at a very low concentration suggested that controlling melanosome transport could be a good target for regulation of skin pigmentation and this compound could be used as a potential skin whitening agent for treating hyper-pigmentation disorders.

## Materials and Methods

### Materials

MNQO was purchased from Green Pharma S.A.S. (France). Phenylthiourea (PTU) was purchased from Sigma-Aldrich Co., St. Louis, MO, USA).

### Cell culture

Melan-a melanocytes are highly pigmented, immortalized murine melanocytes derived from C57BL/6 mice. Melan-a melanocytes were obtained from Dr. Dorothy Bennett (St. George’s Hospital, London, UK). Murine SP-1 keratinocytes, derived from SENCAR mice, were generously provided by Dr. Stuart H. Yuspa (Laboratory of Cellular Carcinogenesis and Tumor Promotion, National Cancer Institute). Melan-a melanocytes were maintained in RPMI-1640 medium (Welgene, Korea) supplemented with 10% fetal bovine serum (FBS), 10 U/ml penicillin, 100 μg/ml streptomycin, 200 nM phorbol 12-myristate 13-acetate (PMA; Sigma-Aldrich Co., St. Louis, MO, USA). The SP-1 keratinocytes were grown in Eagle’s minimal essential medium (EMEM; Lonza) containing 8% Chelex-treated FCS, 100 U/ml penicillin and 100 μg/ml streptomycin.

### Cytotoxicity assay

Melan-a melanocytes were seeded in 96-well plates in RPMI-1640 medium and incubated at 37 °C for 24 h. Each well was then rinsed with DPBS, and DMSO or MNQO was added. After 24 h, 48 h and 72 h, wells were rinsed with DPBS and refilled with medium containing 10% EZ-Cytox (Daeil Lab Service, Korea). After further incubation at 37 °C for 1 h, the optical density of medium in each well was measured at 450 nm using a microplate reader (TECAN, Switzerland).

### Co-culture

Melan-a melanocytes were seeded in six-well plates containing RPMI-1640 medium. Two days later, the plates were rinsed twice with DPBS. The SP-1 keratinocytes were seeded at 1.5 × 10^5^ cells per well to each well containing melanocytes, The initial seeding ratio of keratinocytes to melanocytes was 4:1 and co-cultures of melanocytes and keratinocytes were maintained in EMEM medium (with 0.05 mM Ca^2+^). At 2, 3 and 5 days later, the cells were rinsed with DPBS and resuspended in EMEM (with 0.05 mM Ca^2+^). One day after that, the cultures were photographed.

### Detection and quantification of melanosome aggregation

Melan-a melanocytes were seeded in 24-well plates and maintained for 24 h. The cells were then rinsed in DPBS and treated with samples in RPMI-1640 containing 2% FBS for 3 days. The cells were observed in bright field using an Olympus CKX41 culture microscope (Olympus, Japan), and images were photographed using a DMC camera (INS Industry, Korea) and DMC advanced software adapted to the microscope. Evaluation of melanosome aggregation was performed by counting cells with perinuclear melanosome aggregates in three random microscopic fields per well at x200 magnification. Values are presented as the mean ± SD from three wells (n = 3).

### Maintenance of reconstituted 3-dimensional human skin tissue in culture

Neoderm-ME was purchased from Tego Science, Seoul, Korea, and maintained as instructed by the manufacturer. Neoderm-ME is a viable, reconstituted, 3-dimensional human epidermis containing melanocytes and keratinocytes. In brief, Neoderm-ME was removed from the medium-containing agar and transferred onto 6-well plates for equilibration at 37 °C in 5% CO_2_ for 1 day. The Neoderm-ME was then treated with 1% DMSO and 0.001% MNQO for 7 days. Each compound was dissolved in vehicle and applied directly to the cultured skin surface three times each week, and the tissue samples were kept in an incubator at 37 °C with 5% CO_2_ throughout the course of the experiments. The degree of pigmentation was assessed on photographic images from the brightness value (L value) as determined using editing software (Adobe Photoshop CS; Adobe Systems, USA).

### Measurement of melanin contents

Melan-a melanocytes were seeded in 24-well plates and maintained for 24 h. The cells were then washed in DPBS and treated with samples in RPMI-1640 containing 10% FBS for 3 days. After washing in DPBS, the cells were dissolved in 1 N NaOH at 55 °C for 30 min. The resulting cell lysates were transferred to 96-well plates for optical density (OD) measurements at 490 nm using a microplate reader. For melanin assay using reconstituted 3-dimensional human tissue, Neoderm-ME was dissolved in 1N NaOH and sonicated. The debris was clarified by centrifugation at 13,000 rpm for 5 min, and supernatant ODs were measured at 490 nm using a microplate reader. Protein concentrations of cell lysates were measured using a BCA Protein Assay Reagent Kit (Pierce Biotechnology, Inc, Rockland, IL, USA) with bovine serum albumin as the standard.

### Western blotting

Blots were incubated with β-actin antibody (1:20000, Sigma), MyoVa antibody (1:800, Cell Signaling Technology, Beverly, MA, USA), Rab27a antibody (1:500, Santa Cruz, CA, USA), Mlph antibody (1:600, ProteinTech Group, Inc., Chicago, IL,USA) and MITF antibody (1:1000, Thermo Fisher Scientific, Fremont, USA) at 4 °C overnight. Blots were then washed three times with TBS-T and incubated with horseradish peroxidase-conjugated anti-rabbit (1:1000, Bethyl Laboratories, Montgomery, USA) or anti-mouse (1:20000, Bio-Rad, Hercules, CA, USA) antiserum at room temperature for 1 hour. Bound antibodies were detected using a WEST-ZOL^®^ Plus Western Blot Detection System (INtRON Biotechnology, Korea). The bands on membranes were detected with chemiluminescence and visualized with Chemi Doc XRS (Bio-Rad, USA).

### Quantitative real-time PCR

Quantitative real-time PCR was performed using a FastStart Essential DNA Probe Master kit (Roche, Mannheim, Germany) with the Universal Probe Library (Roche). The reaction was carried out according to the manufacturer’s protocol. The probes for Rab27a (#63, NM_023635.5), Mlph (#108, NM_053015.3), MyoVa (#63, NM_010864.2) and MITF (#27, NM_008601.3) were designed with the Probe Library Assay Design Center. The cycling conditions were 600 s at 95 °C followed by 40 cycles at 95 °C for 20 s and 60 °C for 40 s on Lightcycler^®^ Nano. The resulting cDNA was amplified with the following primers: Rab27a sense 5′-GAAGACCAGAGGGCAGTGAA-3′, and antisense 5′-ACTGGTTTCAAAATAGGGGATTC-3′; Mlph sense 5′-AGCCCCTCAACAGCAAAAA-3′, and antisense 5′-TTCCTCAAAGTCCACATCTCG-3′; MyoVa sense 5′-GCGCCATCACCCTAAACA-3′, and antisense 5′-CCAGTTGACTGACATTGTACCTG-3′; MITF sense 5′-CTAAGTGGTCTGCGGTGTCTC-3′, and antisense 5′-GGTTTTCCAGGTGGGTCTG-3′. Expression levels of the three genes were normalized to mouse β-actin.

### UVB-induced pigmentation in brown guinea pigs

Eight-week-old female brown guinea pigs were supplied by Central Laboratory Animal Inc. (Seoul, Korea). Animal experimental protocol was approved by the ethical committee of Kyung Hee University (no: KHU-12-04) and was carried out in accordance with the approved guidelines. Brown guinea pigs were kept in an air-conditioned room under a temperature 24 °C ± 1 and light/dark cycle at 12 h. After acclimation one week, brown guinea pigs were housed in individual cages. Guinea pigs were anesthetized using ketamine and rompun (4:1 ratio, 1 ml/kg) and five separate areas (1.5 cm × 1.5 cm) on the back of each animal were exposed to UVB radiation (six UVB lamps: TL 20W/12RS, Phillips). The total energy dose of UVB irradiation was 380 mJ/cm^2^ per exposure. Five skin sites from three animals were exposed to the UVB irradiation three times a week for three consecutive weeks. MNQO was then applied topically to the UVB-induced areas once a day for five consecutive weeks. The vehicle (propylene glycol:ethanol:water = 5:3:2) was used as a control. Colorimeter (CR-300, Minolta, Japan) was applied once a week for five consecutive weeks to determine the L value (lightness). The difference of L value was measured:


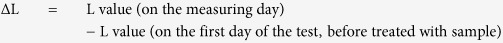


### Immunohistochemical staining

Skin biopsy specimens were taken from the MNQO-treated and vehicle-treated area of guinea pigs after 5 weeks. Biopsied sections were stained with Fontana-Masson method and Hematoxylin and eosin (H&E) method for identified melanin content and skin histological property.

## Additional Information

**How to cite this article**: Park, J. *et al*. Inhibitory effect of 2-methyl-naphtho[1,2,3-de]quinolin-8-one on melanosome transport and skin pigmentation. *Sci. Rep.* **6**, 29189; doi: 10.1038/srep29189 (2016).

## Figures and Tables

**Figure 1:**
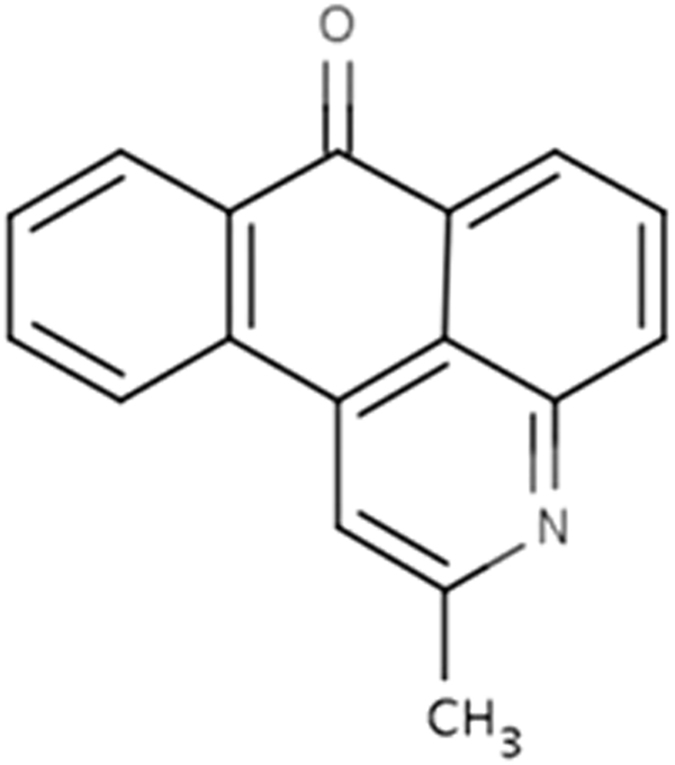
The structure of MNQO.

**Table 1:** The number of aggregated cells per total melanocytes in co-culture.

Well	Fields	24 h			72 h		
		DMSO	2 μM	10 μM	DMSO	2 μM	10 μM
1	#1	0/11	1/12	1/7	0/12	2/14	3/10
	#2	0/9	1/11	2/9	0/10	2/10	4/11
	#3	0/8	1/6	1/9	0/12	1/8	3/13
2	#1	0/12	1/11	2/10	0/10	1/13	3/12
	#2	0/8	0/9	2/8	0/11	1/9	2/13
	#3	0/7	1/10	2/9	0/9	1/8	3/12
3	#1	0/9	1/10	1/8	0/10	2/9	4/11
	#2	0/9	2/9	1/11	0/13	2/11	3/13
	#3	0/8	1/8	1/11	0/9	2/12	4/10
